# The effect of a novel wearable feedback device on cardiopulmonary resuscitation training in medical students: a three-arm cluster randomized controlled trial

**DOI:** 10.3389/fpubh.2026.1883769

**Published:** 2026-07-21

**Authors:** Enze Liu, Liyuan Gan, Wenwen Ma, Mo Chen, Chen Zhang, Xintao Huang, Huisheng Deng

**Affiliations:** 1 Department of General Practice, The First Affiliated Hospital of Chongqing Medical University, Chongqing, China; 2 Chongqing Yihe Integrative Medicine Clinic Co., Ltd., Chongqing, China; 3 Chongqing Liangjiang Newarea and Community Health Service Center, Chongqing, China; 4 Yuzhong District Shangqingsi Subdistrict Community Health Center, Chongqing, China

**Keywords:** cardiopulmonary resuscitation, chest compression, deliberate practice, education, real-time feedback device

## Abstract

**Background:**

While real-time feedback devices are proven to improve skill mastery in CPR training, current options are often suboptimal due to high costs, skin irritation, and single-functionality constraints. To overcome these limitations, we engineered a novel wearable feedback device, the accuracy of which was validated in our prior research. Furthermore, we conceptualized a hybrid training paradigm combining real-time feedback with deliberate practice. Given the current lack of efficacy data for this approach, this study aims to assess the device’s real-world training performance and investigate whether this hybrid training approach can further optimize resuscitation training outcomes.

**Methods:**

Students from Chongqing Medical University were recruited and randomly assigned to three groups: traditional training (Group A), real-time feedback training (Group B), or real-time feedback plus deliberate practice training (Group C). All participants were required to perform 2 min of chest compressions on CPR manikins. The primary outcomes were the proportion of correct compression depth (50–60 mm) and adequate compression rate (100–120 min^−1^). The secondary outcomes were chest compression depth (mm), chest compression rate (min^−1^), and the proportion of correct compression release.

**Results:**

A total of 618 participants were randomly assigned to Group A (*n* = 210), Group B (*n* = 204), and Group C (*n* = 204). The proportion of correct compression depth in Group B was higher than that in Group A (mean [SD], 82.55 [12.67] % vs. 71.54 [22.28] %, mean difference [95% CI], −11.0 [−14.5 to −7.5] %, *p* < 0.001) and lower than that in Group C (90.01 [8.32] %, mean difference, 7.5 [5.4 to 9.6] %, *p* < 0.001). The proportion of adequate compression rate in Group B was higher than that in Group A (mean [SD], 81.61 [14.59] % vs. 78.15 [13.60] %, mean difference [95% CI], −3.5 [−6.3 to −0.7] %, *p* = 0.014) and showed no significant difference from that in Group C (83.12 [11.93] %, mean difference [95% CI], 1.5 [−1.1 to 4.1] %). The compression depth in Group B was deeper than that in Group A (mean [SD], 57.02 [5.15] mm vs. 53.47 (4.71) mm, *p* < 0.001) but did not differ from that in Group C (57.45 [4.36] mm, *p* = 0.367). No significant differences were observed in chest compression rate or the proportion of correct compression release among the three groups (*p* > 0.05).

**Conclusion:**

In conclusion, within the academic setting of a medical university, this novel feedback device improved CPR performance in compression depth and rate among medical students compared to traditional training. Furthermore, the hybrid training method combining real-time feedback and deliberate practice further enhanced training efficacy regarding chest compression depth in this population. However, these findings require independent replication in broader populations and diverse clinical settings before widespread implementation recommendations can be made.

## 1 Introduction

Cardiac arrest (CA), one of the leading causes of death worldwide (1, 2), poses a severe challenge to emergency medical systems due to its abrupt onset and poor prognosis. In Europe, the survival rate for out-of-hospital cardiac arrest (OHCA) is 8%, whereas in China—a populous country—the survival rate for OHCA patients is merely 1% (3, 4), which is significantly lower than the global average (5). As a core emergency intervention, high-quality cardiopulmonary resuscitation (CPR) serves as a critical link in improving patient survival rates, whether performed by laypersons in out-of-hospital settings or emergency physicians in clinical environments (6, 7). The core components of high-quality CPR include minimizing pauses in chest compressions, ensuring adequate compression rate and depth, avoiding chest leaning, and preventing excessive ventilation (8). Therefore, cultivating personnel with standardized CPR operational capabilities is of great practical significance for optimizing the emergency chain of survival and improving patient prognosis (9).

As future healthcare professionals, medical students are required to complete basic life support courses during their academic training. However, relevant studies have shown that even trained professionals often struggle to deliver high-quality chest compressions, which they frequently perform below the recommended standards (10). To improve the quality of CPR operation training, various medical institutions have explored a variety of solutions, among which CPR devices equipped with audio-visual feedback functions have become the focus of attention in the industry, as they can provide real-time assistance in standardizing operations and help learners promptly correct operational deviations (11, 12).

Although current guidelines have highlighted the potential of feedback devices in CPR education (13), previous studies indicate that existing devices possess certain limitations. In response, we report a novel wearable CPR feedback device (the wearable CPR real-time audio-visual feedback device, CPR-BT, ZL 2023 23470890.6) (14). This device not only provides real-time audiovisual feedback during performance but also comprehensively evaluates the trainee’s overall chest compression quality post-training. Leveraging this capability, trainees can enhance their performance through “deliberate practice”—a resuscitation educational strategy endorsed by the American Heart Association (AHA) guidelines (15). This approach integrates repetitive training with assessment-based personalized feedback, enabling the development of targeted training protocols tailored to individual operators’ weaknesses (16).

Additionally, while previous research has confirmed the reliability of data collected by this device (14), its effectiveness in actual training remains unclear and requires further trial verification. Furthermore, while both real-time feedback devices and deliberate practice have demonstrated favorable outcomes in resuscitation education, evidence regarding whether combining these two modalities yields additional improvements in training outcomes remains insufficient. Additionally, pre-specified subgroups were included in the study to explore whether intervention effects vary by sex, weight (17), and learning ability (18).

Therefore, the present study has two primary objectives: first, to investigate whether this novel feedback device can effectively enhance CPR training outcomes compared with traditional training; second, to verify whether the training method combining real-time feedback and deliberate practice can further optimize training outcomes. Our hypotheses are as follows: (1) Compared with traditional training, this novel feedback device can effectively improve CPR training outcomes; (2) The hybrid training integrating real-time feedback and deliberate practice can further enhance training outcomes.

## 2 Methods

### 2.1 Study design

This study was a parallel 1:1:1 cluster randomized controlled trial (RCT) conducted in accordance with the principles of the Helsinki Declaration (19). The study protocol was reviewed and approved by the Ethics Committee of the First Affiliated Hospital of Chongqing Medical University (No.: 2025–650-01) and registered on the Chinese Clinical Trial Registry (ChiCTR2500111177). All participants provided written informed consent.

### 2.2 Participants

This cluster RCT was conducted at Chongqing Medical University from November 2025 to February 2026. Second-year undergraduate students from the First Clinical College of Chongqing Medical University were recruited through class administrators and grouped for training in the clinical skills training classroom of Chongqing Medical University. The inclusion criteria were age ≥ 18 years, voluntary participation, and no prior professional CPR training within the past 2 years. Participants with conditions that precluded the performance of CPR (e.g., upper limb fractures, inability to engage in strenuous exercise) were excluded.

### 2.3 Randomization and blinding

Randomization was performed by an independent statistician using SPSS software. To account for potential differences in academic aptitude reflected by admission scores, a stratified cluster randomization was implemented based on specialty (Clinical Medicine, Anesthesiology, and Psychiatry). Within each specialty stratum, classes (clusters) were allocated to the three intervention groups in equal proportions using a computer-generated random number sequence: the traditional training group (Group A), the real-time feedback training group (Group B), and the combined real-time feedback and deliberate practice training group (Group C). The allocation sequence was generated prior to participant recruitment and strictly concealed to ensure the research team remained blinded to group assignments.

All groups received training from the same senior instructor holding CPR certification, who was not a member of the research team. Outcome assessment for the final 2-min compression test was conducted by independent researchers with CPR qualification credentials. Due to the nature of the interventions, participants could not be blinded to the instructor. However, assessors were blinded to group assignments. To ensure assessor blinding, the following strict isolation procedures were implemented: (1) Automated Data Collection: Primary and secondary outcomes (e.g., compression depth, rate) were automatically recorded by the Laerdal system. Participants were identified solely by their randomly assigned sequence number during testing; (2) Spatial Separation: Training and testing areas were strictly segregated. The instructor immediately left the training room upon completing the session, after which participants proceeded alone to the testing area for the 2-min compression test; (3) Information Isolation: Strict operational protocols were formulated before the commencement of the study, explicitly prohibiting any discussion between instructors and assessors regarding participant group assignments, training performance, or intervention details. Furthermore, instructors explicitly instructed all participants *before testing* that discussing the type of training received or their group assignment was strictly prohibited upon entering the testing room and during the test itself.

### 2.4 Interventions

**Figure 1:**
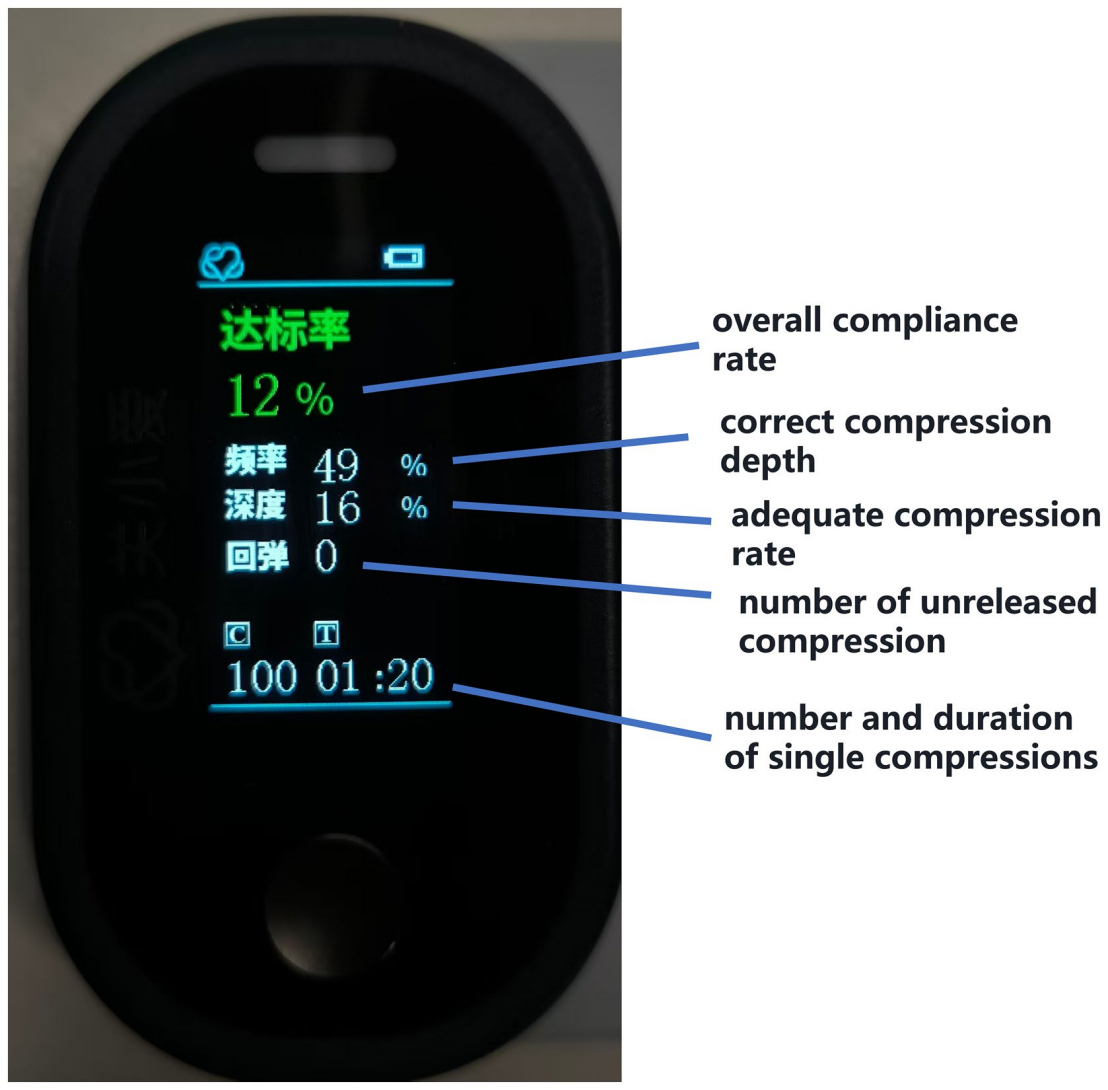
Schematic diagram of compression assessment results.

Training followed the standard Basic Life Support (BLS) course (13). All groups received identical theoretical training, including firstly: (1) CPR concepts, indications, equipment preparation, and precautions; (2) instructor demonstration of skills. Then, skill training commenced. During practice: Group A received traditional CPR training, with the instructor providing face-to-face guidance after each 2-min chest compression practice session. Compared to Group A, prior to practice, participants in Group B first received an equipment briefing from the instructor (how to use the device for practice, interpret the real-time feedback, and make necessary adjustments). During the 2-min chest compression training, they utilized the real-time feedback features of the novel feedback device (screen color, audio prompts, and vibration), allowing for immediate performance adjustments based on the prompts. No instructor feedback was provided following any practice session. Compared to Group B, prior to practice, participants in Group C received the identical equipment briefing from the instructor, along with an introduction to the customized assessment report (Figure 1). During the practice sessions, in addition to real-time feedback, Group C received a customized assessment report immediately following each practice session. The report included: correct compression depth, adequate compression rate, number of unreleased compressions, and overall compliance rate (the proportion of compressions simultaneously achieving adequate depth, appropriate rate, and full chest recoil out of the total number of compressions). The customized report was generated immediately after each 2-min practice cycle, providing participants with 1 min to review it. To operationalize deliberate practice, participants were required to follow a three-step process after each cycle: (1) Identify deficiencies: determine the weakest performance metric in the report; (2) Formulate a strategy: establish a single, targeted improvement goal for the subsequent cycle based on this weakness; (3) Focused execution: concentrate solely on improving that specific parameter during the next 2-min practice cycle. Consistent with Group B, no instructor feedback was provided following any practice session in Group C. Due to the fixed training duration, strict mastery thresholds were not implemented.

It should be noted that, although the overall compliance rate is part of the customized report displayed on the feedback device screen to help students monitor their overall performance and make self-adjustments, the manikin’s data extraction software used during the assessment does not record this metric as an exportable variable in the backend. Due to this objective technical constraint, we were unable to extract this data after the practice assessment, and therefore did not collect it as an outcome measure for statistical analysis. In Figure 1, we present it solely as a core functional feature of the feedback device to illustrate the customized report received by the intervention group.

The total training duration was 180 min (30 min of theoretical training and 150 min of practical training) ensuring sufficient time for deliberate practice. The 150-min hands-on training consisted of: (1) a 10-min demonstration and explanation of key techniques by the instructor; (2) a 140-min chest compression practice. During the practice session, participants were divided into groups of 4 to 6. They took turns performing 2-min compression cycles on the manikins, with rapid rotation within the group after each cycle. Six CPR training manikins (Resusci Mini Anne) were provided to ensure that every participant completed 10 cycles of chest compression practice.

### 2.5 Outcomes

The primary outcomes were the proportion of correct compression depth [defined as compressions with a depth between 50 and 60 mm, expressed as *P*_depth_ = *N*_depth(50 − 60mm)_/*N*_total_ × 100%] and the proportion of adequate compression rate [defined as compressions with a rate between 100 and 120 min^−1^, expressed as *P*_rate_ = *N*_rate(100–120 min_^−1^_)_/*N*_total_ × 100%]. Secondary outcomes included chest compression depth (mm), chest compression rate (min^−1^), and the proportion of correct compression releases [defined as the number of complete chest wall recoil cycles per total compressions, expressed as *P*_release_ = *N*_release_/*N*_total_ × 100% (13)]. Following the training, all participants underwent a 2-min independent simulated chest compression test using Resusci Mini Anne manikins in a standard CPR scenario (20). Measurements were obtained using a certified manikin (Resuscitation Mini Anne; Laerdal Medical), and compression data were collected using the QCPR application (QCPR APP|Laerdal Medical). Data on pressure and displacement were collected in real-time by the QCPR application. The system algorithm defined one complete compression cycle as the interval from the start of compression (detected when pressure threshold was reached) to full chest wall recoil. Compression rate was calculated based on the time interval between consecutive compressions. At the end of the 2-min test, the system automatically aggregated data from all individual compressions and outputted the final results.

To avoid measurement circularity, the measurement accuracy of the wearable device was validated through independent external testing (Report No. WT241682). This validation was independent of the Laerdal system, using an electronic stopwatch to measure compression rate and a laser distance meter to measure compression depth. The complete validation report is available in Supplementary File 1.

### 2.6 Sample size estimation

According to previous reports, the proportion of appropriate chest compression depth was approximately 40% without feedback devices and approximately 70% with feedback devices (11). We assumed the proportion for the real-time feedback combined with deliberate practice group was 85%. Given that detecting a smaller effect size requires a larger sample size, we based our sample size estimation on the comparison between Groups B and C to ensure sufficient statistical power for the study. Based on empirical data from healthcare cluster randomized trials (21) and the implications of our study design—specifically, that the primary outcome is an objective psychomotor skill measured individually via manikins (22)—we assumed an intraclass correlation coefficient (ICC) of 0.02. With an average cluster size of 30, a two-sided *α* level of 0.05, and 80% statistical power, the required sample size was calculated to be 576.

### 2.7 Statistical analysis

All statistical analyses were performed using IBM SPSS Statistics 27.0. Normality and homogeneity of variance were first assessed for all continuous variables. Depending on their distribution, continuous variables were presented as mean ± standard deviation (SD) or median (interquartile range, IQR). For normally distributed continuous variables with homogeneous variances, one-way analysis of variance (ANOVA) was used for comparisons among the three groups, followed by Tukey’s *post hoc* test for pairwise comparisons; in cases of unequal variances, Welch’s ANOVA was employed, with Games–Howell *post hoc* test for pairwise comparisons. For non-normally distributed continuous variables, the Kruskal–Wallis test was used for group comparisons; if the overall difference was statistically significant, Dunn’s test was performed for pairwise comparisons with Bonferroni correction for multiple comparisons. Categorical variables were presented as frequencies (percentages) and compared using the Chi-square test; Fisher’s exact test was used when the expected frequencies violated the assumptions of the Chi-square test. If the overall comparison was statistically significant, pairwise comparisons were further conducted with Bonferroni correction. Given that this study was designed as a cluster randomized controlled trial with randomization performed at the class level, observations among individuals may exhibit non-independence (clustering effect). Therefore, the clustering effect was accounted for in all primary analyses. For continuous variables meeting the assumptions of normality and homogeneity of variance, linear mixed models (LMMs) were used for comparisons, with the intervention group as a fixed effect and the class as a random effect (random intercept) to control for the clustering effect. If the overall comparison yielded a statistically significant difference, post-hoc pairwise comparisons were performed based on the model-estimated marginal means, with the Bonferroni method applied to adjust for multiple comparisons. For continuous variables violating the assumptions of parametric tests (e.g., non-normal distribution or heteroscedasticity), analyses were conducted using LMMs following appropriate data transformation, LMMs with robust standard errors, or generalized linear mixed models (GLMMs), depending on the data distribution characteristics. Post-hoc pairwise comparisons were similarly adjusted using the Bonferroni method.

Additionally, subgroup analyses were performed based on variables such as sex, weight category, and specialty to evaluate the differences in intervention effects across populations with different characteristics, along with tests for interaction effects. For continuous outcome variables, linear models including an interaction term or two-way ANOVA were employed when the assumptions of parametric tests were met—including independent observations, approximately normal distribution of model residuals, and homogeneity of variance. In these models, the outcome measure served as the dependent variable, while the intervention group, the subgroup factor, and their interaction term served as independent variables. If these parametric assumptions were violated, analyses were conducted using robust standard errors, bootstrapping, appropriate data transformations, generalized linear models, or other robust methods, depending on the distribution of the outcome variable. The *P* for interaction was used to assess whether the intervention effect differed significantly across subgroups. For normally distributed outcomes, results were expressed as mean difference (95% CI); for non-normally distributed outcomes, median differences (95% CI) were calculated using the Hodges-Lehmann Estimator method (23). A *p-*value <0.05 was considered statistically significant.

## 3 Results

### 3.1 Participant flow and recruitment

**Figure 2:**
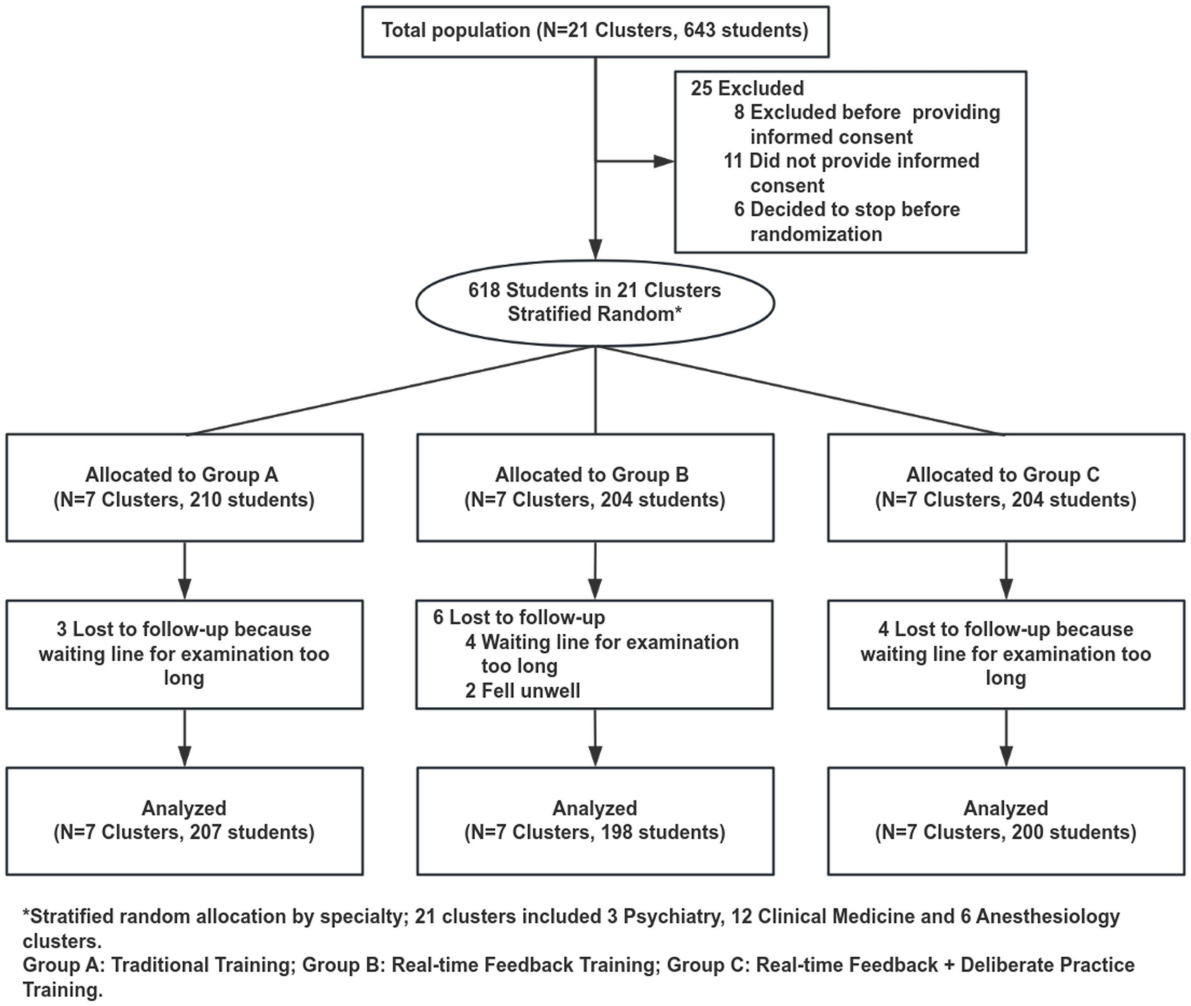
CONSORT flow diagram.

A total of 21 clusters were included in the study, with 632 informed consents provided (The allocation and sizes of each cluster are detailed in Supplementary Table 1). Prior to the initiation of randomization, 14(2.2%) were excluded, including 8 participants who failed to meet the inclusion criteria and 6 participants who voluntarily withdrew. A total of 618 participants from 21 clusters were randomly assigned: 7 clusters (212 participants) were randomized to traditional training (Group A), 7 clusters (207 participants) to real-time feedback training (Group B), and 7 clusters (205 participants) to real-time feedback plus deliberate practice training (Group C). After the training session, 13 participants did not participate in the subsequent assessment (2 due to physical discomfort and 11 due to excessive waiting time). Ultimately, a total of 605 participants (97.9%) completed the outcome assessment (Figure 2).

### 3.2 Baseline data

Among the 605 participants, 374(61.8%) were female and 231(38.2%) were male. The mean (SD) age was 19.64 (0.94) years, the median (interquartile range [IQR]) weight was 58 (43–78) kg, and the mean (SD) grade point average (GPA) was 2.96 (0.74) (Table 1). No statistically significant differences in baseline characteristics were observed among the three study groups.

**Table 1:** Characteristics of study participants at baseline.

Characteristic	No. (%)				*P-*value
	Total *N* = 605	Group A *n* = 207(34.2)	Group B *n* = 198(32.7)	Group C *n* = 200(33.1)	
Age, mean (SD), y	19.64(0.94)	19.58(0.72)	19.69(0.56)	19.62(0.66)	0.967
Sex					
Men	231(38.2)	72(34.8)	78(39.4)	81(40.5)	0.470
Women	374(61.8)	135(65.2)	120(60.6)	119(59.5)	
Weight, median (IQR), kg	58(55–64)	57(54–62)	59(55–64)	59(55–64)	0.905
Major					
Psychiatry	74(12.2)	27(13.0)	26(13.1)	21(10.5)	0.932
Clinical Medicine	366(60.5)	124(59.9)	119(60.1)	123(61.5)	
Anesthesiology	165(27.3)	56(27.1)	53(26.8)	56(28.0)	
GPA	2.96(0.74)	2.89(0.58)	3.02(0.55)	2.95(0.63)	0.574

SD, standard deviation; IQR, interquartile range; GPA, grade point average.

Group A: traditional training; Group B: real-time feedback training; Group C: real-time feedback plus deliberate practice training.

### 3.3 Primary outcomes

The mean (SD) proportion of correct compression depth in Group B (real-time feedback training) was 82.55 (12.67) %, which was higher than that in Group A (traditional training) at 71.54 (22.28) % but lower than that in Group C (real-time feedback plus deliberate practice training) at 90.01 (8.32) %. The mean difference (95% CI) between Groups A and B was −11.0 (−14.5 to −7.5) %, and that between Groups C and B was 7.5 (5.4 to 9.6) %. Group B performed significantly better than Group A (*p* < 0.001) but worse than Group C (*p* < 0.001).

**Figure 3:**
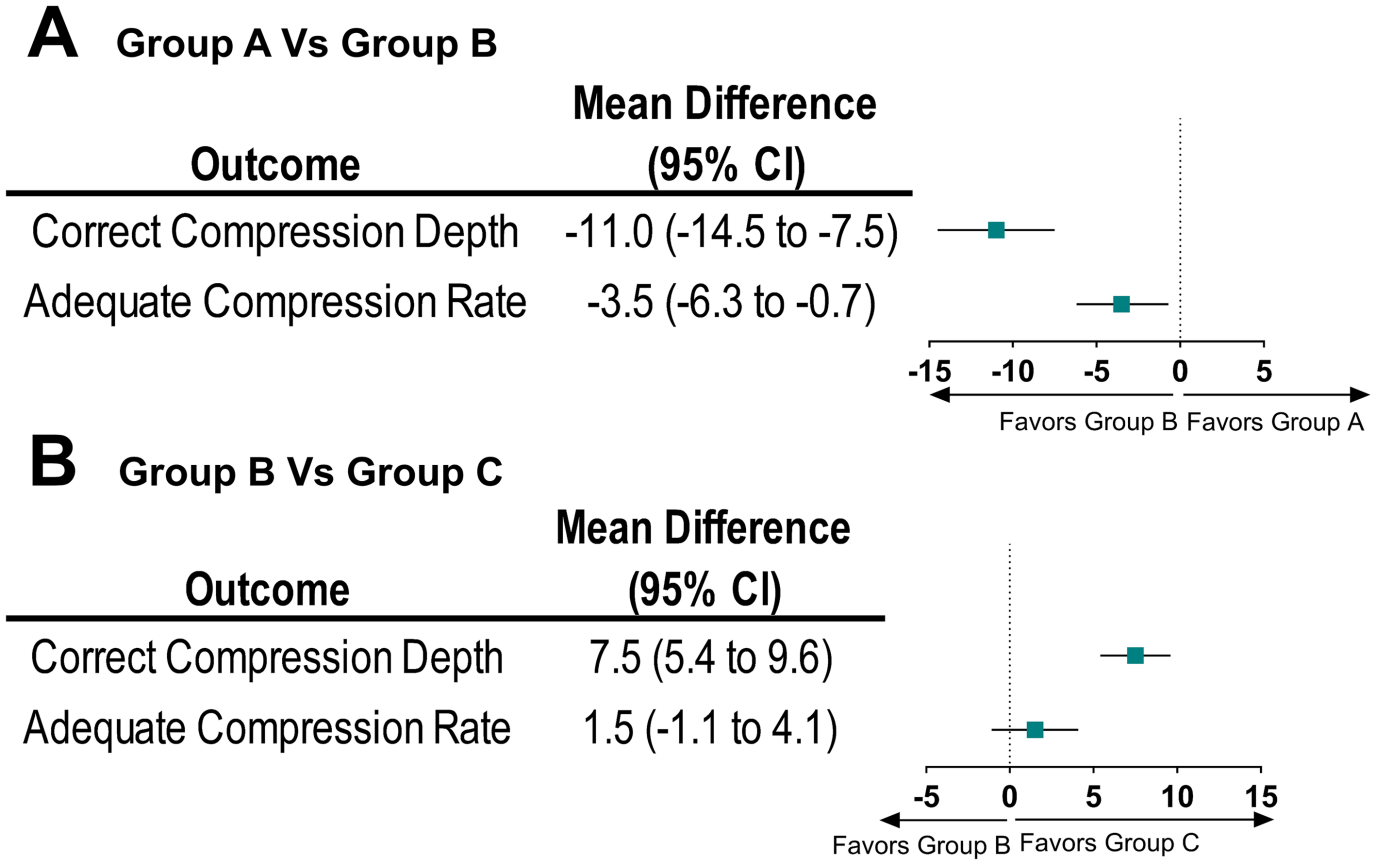
Forest plot analysis of the main results. **(A)** Forest plot of the mean differences (95% CI) in correct compression depth (%) and adequate compression rate (%) between Group A (traditional training) and Group B (real-time feedback training). **(B)** Forest plot of the mean differences in correct compression depth and adequate compression rate between Group B and Group C (real-time feedback + deliberate practice training), with 95% confidence intervals. The dashed line represents the null line.

Additionally, the mean (SD) proportion of adequate compression rate in Group B was 81.61 (14.59) %, which was higher than that in Group A at 78.15 (13.60) % but also lower than that in Group C at 83.12 (11.93) %. The mean difference (95% CI) between Groups A and B was −3.5 (−6.3 to −0.7) %, and that between Groups C and B was 1.5 (−1.1 to 4.1) %. Group B also performed better than Group A (*p* = 0.014), but there was no significant difference between Groups B and C (*p* = 0.259) (Figure 3).

### 3.4 Secondary outcomes

**Figure 4:**
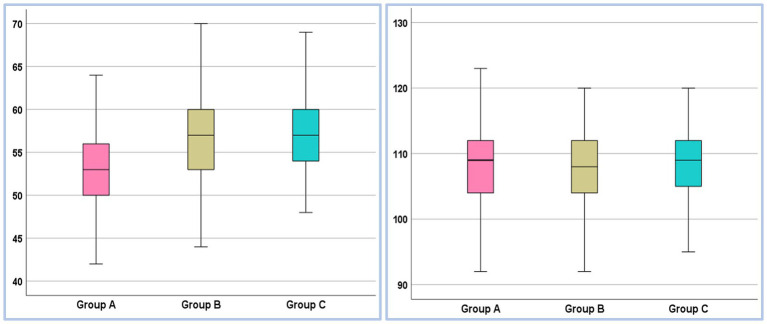
Box plots of compression depth (mm, left) and compression rate (min^−1^, right) among Group A (traditional training), Group B (real-time feedback training), and Group C (real-time feedback + deliberate practice training).

The mean (SD) chest compression depth in Group B was 57.02 (5.15) mm, which was significantly deeper than the 53.47 (4.71) mm observed in Group A (*p* < 0.001) but did not differ significantly from the 57.45 (4.36) mm in Group C (*p* = 0.367). The mean (SD) chest compression rate was 108.26 (6.15) min^−1^ in Group A, 107.98 (5.59) min^−1^ in Group B, and 108.12 (5.47) min^−1^ in Group C, with no significant differences detected among the three study groups (*p* = 0.893). The comparison of chest compression data distributions across the three groups is shown in Figure 4. Additionally, the median [interquartile range (IQR)] proportion of correct compression release in Group B was 98 (86–100) %, which did not differ significantly from 96 (83–100) % in Group A or 98 (89–99) % in Group C (*p* = 0.370).

### 3.5 Ancillary analyses

**Figure 5:**
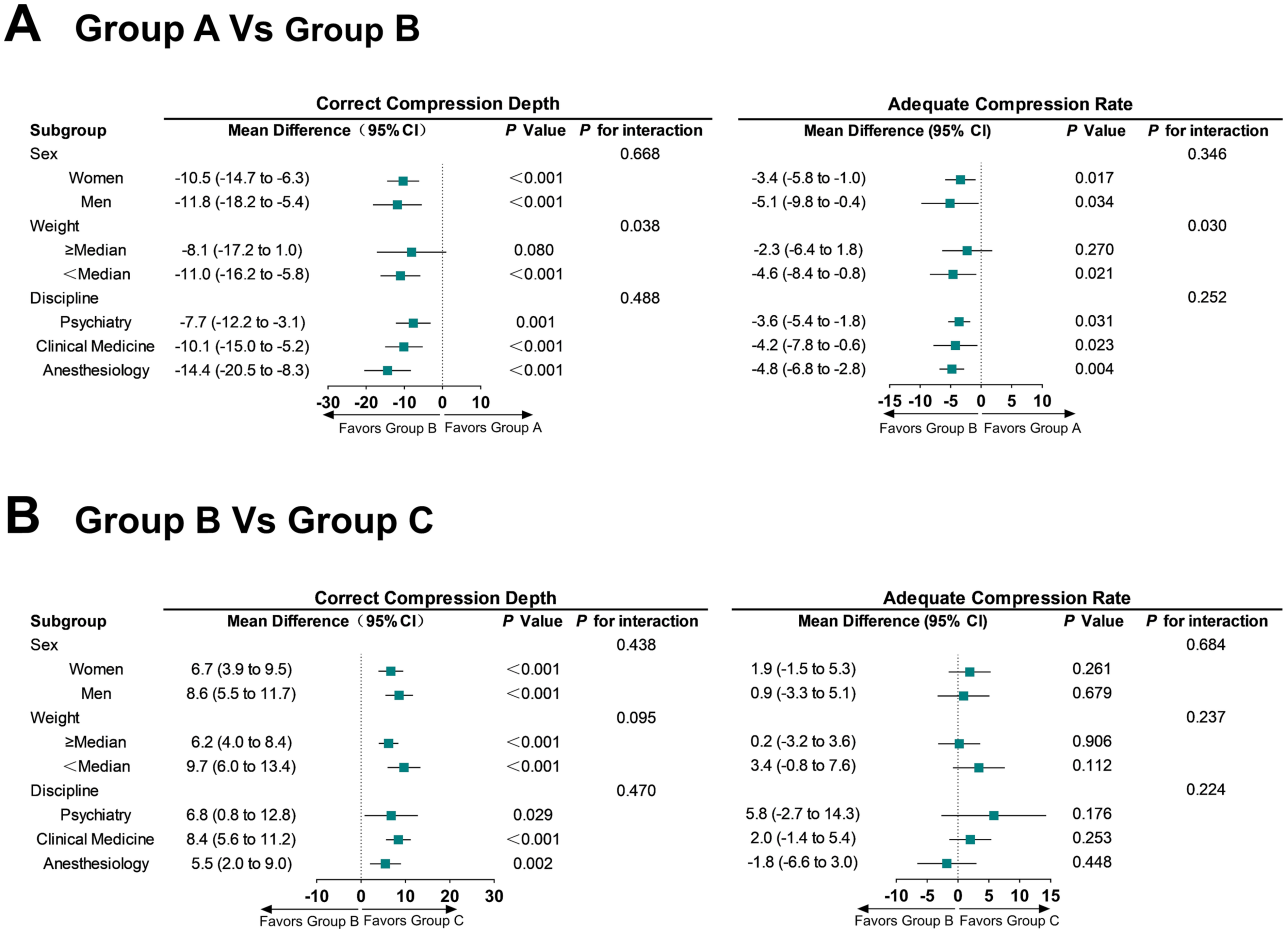
Forest plot analysis of pre-specified subgroups stratified by gender, body weight, and academic major. **(A)** Mean differences (95% CI) in correct compression depth (%) and adequate compression rate (%) between Group A (traditional training) and Group B (real-time feedback training). **(B)**: Mean differences in correct compression depth (%) and adequate compression rate (%) between Group B and Group C (real-time feedback + deliberate practice training), with 95% confidence intervals. *p*-values for interaction were used to test whether the effects of the intervention on correct compression depth and adequate compression rate differed between different subgroups. The dashed line represents the null line.

Subgroup analysis results are shown in Figure 5. The results indicated that heterogeneity in the effects of traditional training versus real-time feedback training on the proportion of correct compression depth (*p* = 0.038 for interaction) and adequate compression rate (*p* = 0.030 for interaction) was observed in terms of weight. The difference between traditional training and real-time feedback training was significantly smaller in the subgroup with weight ≥ median, whereas no significant difference was observed in the other groups. Compared with real-time feedback, the intervention effect of the real-time feedback + deliberate practice training method showed no significant interaction across different subgroups.

## 4 Discussion

In this randomized trial, final assessment results were collected from 605 s-year medical students across 21 clusters at Chongqing Medical University. The study demonstrated that: (1) CPR training using the feedback device improved medical students’ performance in compression depth and rate compared with traditional training; (2) The combination of real-time feedback and deliberate practice further improved chest compression depth, but yielded no additional improvement in compression rate. These findings validate the effectiveness of the feedback device in CPR training and provide important evidence for the application of the “real-time feedback + deliberate practice” training method.

### 4.1 CPR quality with feedback devices

Regarding the comparison between real-time feedback and traditional training, the results showed that real-time feedback training was superior to traditional training in terms of the proportion of correct compression depth and adequate compression rate. This demonstrates that training based on this novel feedback device improved trainees’ chest compression performance more effectively than traditional instructor-led training. These findings are consistent with those of previous studies (24–26).

Additionally, the rapid decay of CPR skills remains a significant challenge in the training field; the pass rates for key metrics, such as compression depth and rate, typically deteriorate 3 to 6 months after initial training (27–30). Therefore, retraining is essential. A study regarding CPR retraining indicated that device-based real-time visual feedback serves as an effective quality assurance method and represents a feasible option for CPR recertification programs (31). For medical students, although they benefit from access to manikins and equipment within medical institutions that allows for repeated practice, the limited availability of faculty often results in a lack of professional guidance during retraining, making it difficult to meet the demands of large-scale self-directed training (32). The use of feedback devices offers a low-cost and feasible solution for CPR skill retraining among medical students in the future (25, 33).

### 4.2 Efficacy of the hybrid training combining real-time feedback and deliberate practice

The results indicate that combining the two training modalities provides additional benefits for chest compression depth. According to the classic “guidance hypothesis” in motor learning (34), concurrent immediate feedback often impedes long-term learning by inducing learner dependence. While the use of real-time feedback in CPR training can improve immediate compression quality, performance frequently declines significantly once the feedback is withdrawn (27). The 2021 European Resuscitation Council Guidelines for Education also explicitly state that device feedback alone is insufficient for long-term skill retention (35). Unlike Group B, Group C received summary feedback after practice and engaged in deliberate practice using a three-step method (identify-strategy-execute) (15). Furthermore, Duvivier et al. (36) confirmed in medical education that such post-practice reflective feedback promotes deeper cognitive processing and skill transfer more effectively than immediate correction. As emphasized by the foundational literature on deliberate practice theory, high-quality training is not merely blind repetition; rather, it requires a highly cognitively engaging process characterized by sustained attention and strategic adjustments (37).

The study demonstrated that a training method combining real-time feedback and deliberate practice can further enhance compression depth. However, implementing deliberate practice is time-consuming in real-world settings. In time-constrained scenarios such as emergency training or public training, brief training utilizing feedback can rapidly enhance core performance quality (38); whereas in time-sufficient settings such as routine medical education, the “feedback + deliberate practice” model should be prioritized to achieve precision improvement, particularly regarding key metrics like compression depth. This aligns with the recommendations of the CPR guidelines (15), which explicitly state that instructional strategies should be tailored to different populations. As a high-intensity, goal-oriented training method, deliberate practice can maximize the utility of feedback data.

However, in terms of secondary outcomes, there was no significant difference in mean chest compression depth between the two groups. This appears inconsistent with the primary outcomes, a finding similar to that of a previous study (39). We explored this discrepancy and, through an analysis of the primary outcome (proportion of correct compression depth), found that although the mean compression depths in both groups were within the guideline-recommended range, the proportion of individual compressions falling within the 50–60 mm range was significantly lower in the real-time feedback group compared to the real-time feedback plus deliberate practice group. Relevant evidence is provided by a study on rescuer fatigue (17), which indicated that as compression duration increases, chest compression depth decreases due to muscle fatigue in the rescuer. This implies that while the final mean compression depth may meet guideline requirements, chest compressions performed during a state of muscle fatigue often fail to achieve the specific standards. A similar pattern was observed regarding compression rate performance. Therefore, mean compression depth and mean compression rate may not accurately reflect actual chest compression performance; however, previous similar studies have frequently used these as primary outcome measures (30, 40–42). Currently, there is no evidence regarding whether the application of these different outcome measures impacts learner clinical performance or patient outcomes. Future studies should further investigate and report on these specific effects.

The results also indicate that there were no differences among the three groups regarding the proportion of correct compression release, though all exceeded 90%. In a brief traditional CPR training study, the proportion of compressions with full release reached 88% (40). This demonstrates that participants can master chest compression release within a relatively short time under instructor guidance.

### 4.3 Instructor role: from assessor to facilitator

In traditional instruction, instructors assess trainees’ performance based on experience and provide subsequent guidance. However, a study by Cheng et al. highlighted the inadequacy of healthcare providers’ subjective judgments regarding CPR quality (26). This suggests that the feedback trainees receive from instructors may be inaccurate, whereas feedback devices can eliminate the assessment biases inherent in traditional teaching through objective metrics. Furthermore, Brown et al. (43) demonstrated that although performing CPR with feedback devices increases the operator’s workload, it simultaneously enhances compression quality. The use of feedback devices during resuscitation can mitigate the diminished perception of CPR quality caused by muscle fatigue. Therefore, the implementation of feedback devices not only improves the accuracy of instructional feedback but also shifts the workload of monitoring compression rate, depth, and full chest recoil from the instructor to the trainee. Consequently, instructors can redirect their focus toward more macro-level guidance—such as overall body mechanics and core engagement—thereby enhancing overall teaching efficiency.

### 4.4 Prespecified subgroup analyses

Subgroup analysis results indicated that compared with traditional training, the impact of training using feedback devices was not significant for participants with higher body weight. A possible explanation is that individuals with higher body weight can leverage their own weight in conjunction with gravity to generate the pressure required for chest compressions, resulting in stable compression quality and lower fatigue (44). This implies that for heavier participants, traditional training is sufficient to meet the requirements for basic operational proficiency. Although stratified randomization was performed prior to the study to minimize the influence of different majors, subgroup analysis revealed no heterogeneity in the effect of majors on the rates of correct compression depth and adequate compression rate when comparing traditional training with feedback training, or feedback training with feedback combined with deliberate practice.

### 4.5 Limitations

Several limitations to our study should be acknowledged. First, the study population consisted exclusively of medical students; thus, the efficacy of this approach among laypersons remains unknown. Future studies should apply this device and the “real-time feedback plus deliberate practice” training modality to CPR training among diverse populations to verify the generalizability of our findings. Second, this study only evaluated immediate training outcomes and did not assess the impact on long-term skill retention. Future research should incorporate follow-up assessments to explore the sustained effects of the device and this novel training paradigm on long-term skill retention. Third, limited by our data extraction system, this study did not record granular data from each training round to construct learning curves. Although the final assessment results strongly confirmed Group C’s advantage in compression depth, the lack of dynamic training process data limits further exploration of the specific evolutionary trajectory of skill internalization. Future studies should employ real-time data capture to plot learning curves to further elucidate this mechanism. Fourth, despite the third-party validation of the device, using the Laerdal system for final outcome assessment still carries unavoidable inherent systematic errors.

## 5 Conclusion

In conclusion, within the academic setting of a medical university, this novel feedback device improved CPR performance in compression depth and rate among medical students compared to traditional training. Furthermore, the hybrid training method combining real-time feedback and deliberate practice further enhanced training efficacy regarding chest compression depth in this population. However, these findings require independent replication in broader populations and diverse clinical settings before widespread implementation recommendations can be made.

## Data Availability

The original contributions presented in the study are included in the article/Supplementary material, further inquiries can be directed to the corresponding authors.
