# Supplementary material for: The effect of a novel wearable feedback device on cardiopulmonary resuscitation training in medical students: a three-arm cluster randomized controlled trial
Source: Front Public Health. 2026 Jul 21;14:1883769. doi: 10.3389/fpubh.2026.1883769 (PMC13433616; doi:10.3389/fpubh.2026.1883769)
Supplement: Supplementary file 1 [file Data_Sheet_1.PDF]

Supplementary File 1: Chongqing Medical Device Quality Inspection Center (Report No.: WT241682)

*(Note: The original official report WT241682 is in Chinese. The following is a detailed English summary of its methodology and findings.) *

1. Purpose and Independence

The purpose of this validation is to evaluate the measurement accuracy of the wearable cardiopulmonary resuscitation (CPR) feedback device regarding compression rate and depth. The methodology and reference standards utilized are strictly independent of the Laerdal system, which was used for outcome assessment in this clinical trial.

2. Methodology

- Compression Rate Evaluation: Researchers used an electronic stopwatch (Tianfu PC894, Shenzhen, China) to track the number of compressions within the range of 80 to 140 compressions per minute. Measurements were taken at 1-minute intervals. One researcher was responsible for timing, while two other researchers (one of whom acted as the rescuer) precisely recorded the compression count. The actual compression rate was then calculated and compared with the average rate recorded by the wearable device during the same period.
- Compression Depth Evaluation: An independent laser distance meter (Qinmai, Shenzhen, China) was used to measure the actual compression depth. The measured physical depth was directly compared with the depth readings recorded by the device.

3. Results

- Compression Rate: Within the range of 80 to 140 compressions per minute, the detection error for compression rate of the CPR assistive device did not exceed ±2 compressions per minute.
- Compression Depth: Within the range of 3 cm to 7 cm, the detection er ror for compression depth of the CPR assistive device did not exceed ± 5 mm.
