# Supplementary material for: The effect of a novel wearable feedback device on cardiopulmonary resuscitation training in medical students: a three-arm cluster randomized controlled trial
Source: Front Public Health. 2026 Jul 21;14:1883769. doi: 10.3389/fpubh.2026.1883769 (PMC13433616; doi:10.3389/fpubh.2026.1883769)
Supplement: Supplementary file 2 [file Table_1.DOCX]

| **Supplementary table 1-**The range and distribution of cluster sizes across groups | | |
| --- | --- | --- |
| **Cluster** | **Group** | **cluster sizes** |
| 1 | Group A | 32 |
| 2 | Group B | 27 |
| 3 | Group B | 30 |
| 4 | Group C | 32 |
| 5 | Group C | 32 |
| 6 | Group A | 31 |
| 7 | Group B | 30 |
| 8 | Group A | 29 |
| 9 | Group C | 30 |
| 10 | Group B | 32 |
| 11 | Group A | 32 |
| 12 | Group C | 27 |
| 13 | Group A | 27 |
| 14 | Group B | 26 |
| 15 | Group C | 21 |
| 16 | Group B | 29 |
| 17 | Group A | 28 |
| 18 | Group C | 32 |
| 19 | Group C | 28 |
| 20 | Group B | 24 |
| 21 | Group A | 28 |
